# Mechanical and Tribological Properties of Ta-N and Ta-Al-N Coatings Deposited by Reactive High Power Impulse Magnetron Sputtering

**DOI:** 10.3390/ma15093354

**Published:** 2022-05-07

**Authors:** Valentina Zin, Francesco Montagner, Silvia Maria Deambrosis, Cecilia Mortalò, Lucio Litti, Moreno Meneghetti, Enrico Miorin

**Affiliations:** 1 Institute of Condensed Matter Chemistry and Technologies for Energy—ICMATE, National Research Council—CNR, Corso Stati Uniti 4, 35127 Padova, Italy; valentina.zin@cnr.it (V.Z.); francesco.montagner@cnr.it (F.M.); cecilia.mortalo@cnr.it (C.M.); enrico.miorin@cnr.it (E.M.); 2 Dipartimento di Scienze Chimiche, Università degli Studi di Padova, Via F. Marzolo 1, 35131 Padova, Italy; lucio.litti@unipd.it (L.L.); moreno.meneghetti@unipd.it (M.M.)

**Keywords:** tantalum nitride, tantalum aluminum nitride, high-power impulse magnetron sputtering, mechanical properties of films, tribological properties, wear resistance

## Abstract

In this article, the depositions and functional characterizations of Ta-N and Ta-Al-N coatings for protection purposes, grown by reactive high-power impulse magnetron sputtering onto silicon substrates, are described. Nitride films were grown while changing the substrate polarization voltage (i.e., the applied bias voltage) during the process. Moreover, the effects of adding Al to form a ternary system and the resulting variation of the coatings’ mechanical and tribological properties have been widely investigated by nanoindentation, scratch, and wear tests. Micro-Raman characterization has been applied to the wear tracks to explore the comprehensive tribo-environment and wear mechanism. Interestingly, Ta-Al-N films, despite significantly improved mechanical properties, show a premature failure with respect to Ta-N coatings. The wear mechanisms of Ta-N and Ta-Al-N systems were revealed to be very different. Indeed, Ta-Al-N films suffer higher oxidation phenomena during wear, with the formation of an oxidized surface tribofilm and a reduced wear resistance, while Ta-N coatings undergo plastic deformation at the wear surface, with a slightly adhesive effect.

## 1. Introduction

Coating solutions with high wear resistance, improved mechanical properties, and upgraded friction behavior are ever more in demand, thanks to recent and growing advances in modern manufacturing technologies for thin film deposition on increasingly diversified substrates [1,2]. In the last few decades, the protective capabilities of transition metal nitride coatings deposited by reactive magnetron sputtering have been extensively investigated. However, most studies focus on a restricted number of elements, such as Cr, Ti, and Al, while other potentially interesting compounds are seldom considered [3,4,5].

Nevertheless, the industry is constantly demanding coatings with improved properties. Hence, research is very active in the progress of structures, materials, and deposition technologies that are able to withstand the ever more severe working conditions encountered in actual applications.

Tantalum forms binary and ternary nitrides; these have recently been widely investigated in relation to their electric and thermal properties, especially in the microelectronic sector. Until now, little work has been conducted into the tribological behavior of Ta-N-based coatings, despite the fact that tantalum nitride is a hard material, one that is chemically inert and corrosion-resistant [6,7,8], thus being attractive for many applications, including in the mechanical industries in the form of compressor blades and gearboxes, or in the form of coated tools, such as for high-speed cutting and drilling [9]. With the aim of increasing the service life of tools, work efficiency, and reducing costly downtimes, the protection of cutting-tool materials by means of the application of a hard and wear-resistant coating is a promising route.

Moreover, tantalum nitride also exhibits great potential for chemical devices and medical implants [10,11,12], since tantalum exhibits a corrosion performance similar to that of noble metals, being among the most corrosion-resistant materials. In addition, it is not attacked by acids and is biocompatible [7,13,14]. The notable chemical resistance of tantalum and its nitrides depends on the highly stable and well-adherent native tantalum oxide (Ta_2_O_5_) film forming on the surface, even under normal exposure to the atmosphere [15]. Tantalum binary and ternary nitrides are also extensively investigated in the form of diffusion barriers for metallization systems made of copper [16,17] since they can withstand high temperatures, act as resistors in electronic and optical devices [18,19,20], and as film resistors in integrated circuits [21].

Ta-N coatings can be deposited by atomic layer deposition (ALD) and ion beam-assisted deposition (IBAD), as well as via chemical (CVD) and physical (PVD) vapor deposition techniques [7,8,22]. Among them, reactive sputter deposition is favored and offers the opportunity to tailor the film’s properties within a wide range [23]. The Ta-N system is quite complex and provides numerous related structures that slightly differ in terms of N_2_ content during sputtering, as well as changes in pressure, temperature, and deposition duration [24]. Besides the known equilibrium phases of bcc α-Ta, solid–solution α-Ta(N), hexagonal γ-Ta_2_N, and hexagonal ε-TaN, several metastable phases have been observed, including tetragonal β-Ta, bcc β-Ta(N), hexagonal Ta_2_N, the hexagonal WC-structure θ-TaN, the cubic B1 NaCl-structure δ-TaN, hexagonal Ta_5_N_6_, tetragonal Ta_4_N_5_, and orthorhombic Ta_3_N_5_ [24,25]. The formation of such metastable phases is a typical effect of the nitriding kinetics of tantalum, which depends on the inter-solubility of the Ta matrix, with a small amount of distorted N solution phase having a different lattice structure [26]. Thus, careful attention needs to be paid while depositing Ta-N-based coatings by magnetron sputtering technologies, since the properties of final products are associated with specific crystal structures that are strongly affected by selected process parameters.

Conventional direct current magnetron sputtering (DCMS) is one of the state-of-art techniques for the production of hard coatings, e.g., Ta-N-based ones, but obtained sputtered films are typically affected by numerous defects that strongly compromise their performance. To overcome this issue, the implementation of high-power impulse magnetron sputtering (HiPIMS) technology can be exploited [27]. HiPIMS provides pulsed magnetron sputtering, characterized by the generation of ultra-dense plasma near the target surface, which leads to a high degree of ionization of the sputtered atoms. This allows the depositing of adherent, exceptionally dense and smooth coatings, including on complex-shaped substrates [28,29].

In this work, the deposition of both binary Ta-N and ternary Ta-Al-N nitrides for protective purposes has been implemented by means of HiPIMS technology. Ternary nitrides are generally achieved by adding a third material, such as V, Al, Si, Mo, or Zr to a binary nitride compound in order to modify the morphology, structure, and bonding of the coating [30,31,32]. The addition of a certain element to a binary system could provide improved oxidation resistance, chemical stability, wear resistance, and high-temperature hardness retention [33]. Here, Al was selected to investigate the formation of a ternary system and the consequent variation of mechanical and tribological properties of the coatings for protection purposes. Two series of Ta-N and Ta-Al-N coatings, with a maximum thickness of 3 µm, were deposited by varying the bias applied to conductive doped Si (100) substrates during the HiPIMS deposition process. The final products were analyzed, in terms of structural and mechanical/tribological properties, by means of X-ray diffraction, scratch and wear tests, nanoindentation, and µ-Raman analyses of the worn surfaces.

## 2. Materials and Methods

### 2.1. Film Deposition

**Figure 1:**
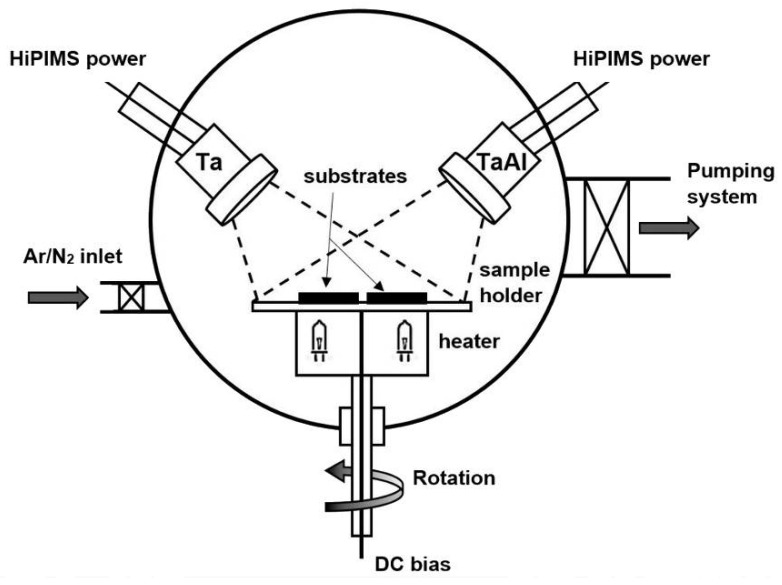
Schematic of the deposition chamber setup.

Binary tantalum nitride (Ta-N) and ternary tantalum aluminum nitride (Ta-Al-N) films were deposited via reactive high-power impulse magnetron sputtering (Re-HiPIMS) on Si(100) substrates. 99.99% Ta and 99.95% powder sintered TaAl (Ta:Al = 1:1 atomic ratio) targets, 100 mm in diameter, mounted on slightly unbalanced magnetron cathodes (MAK 4”, Meivac Inc., San Jose, CA, USA) were used as source materials. Silicon substrates were washed in acetone/isopropyl alcohol mixed solution in an ultrasonic bath, then rinsed with acetone and, finally, dried with pure nitrogen. Before the depositions, the chamber was evacuated to a base pressure lower than 1 × 10^−4^ Pa, using a turbomolecular pump and a rotary vane pump. During each deposition, a mixed gas atmosphere composed of inert Ar (99.9997% purity, Nippon Gases Italia, Milano, Italy) and reactive N_2_ (99.999% purity, Nippon Gases Italia, Milano, Italy) was introduced through dedicated mass flow controllers (MKS). The total gas pressure was kept constant at 1 Pa. The deposition temperature was set at 200 °C by preheating the substrate with a halogen lamp array; it was monitored by two type-K thermocouples placed as close as possible to the samples. The sample-holder was rotated by a motorized system to promote coating uniformity. The two sputter sources used were inclined in order to focus plasma on the center of the substrate holder, as shown in Figure 1.

The target-to-substrate distance was fixed at 120 mm for all sputtering runs. The coatings were deposited using a True Plasma High Pulse 4002 power supply (TRUMPF Hüttinger, Freiburg, Germany). The average sputtering power density was set at 6.4 W/cm^2^, with a pulse frequency of 500 Hz and pulse length of 25 µs. When using the tantalum source, the peak current density J_pk_ was about 1.7 A/cm^2^ and the peak voltage, V_pk_ ~ 850 V for all depositions: J_pk_ ~ 1.0 A/cm^2^ and V_pk_ ~ 930 V when sputtering the TaAl target. Deposition durations were optimized in order to obtain films of comparable thicknesses. A negative direct current polarization was applied to the substrate using a 3018 HBP bias unit (TRUMPF Hüttinger, Freiburg, Germany). Different bias values were used to investigate their effects on the final properties (i.e., −50 V, −75 V, and −100 V). To promote adhesion and mechanical support an inter-layer of Ta-Al alloy was firstly deposited under the same conditions on all the substrates. After film deposition, the samples were kept under a vacuum at 200 °C for 1 h and then left to cool down to room temperature before their extraction from the chamber.

### 2.2. Film Characterization

Energy dispersive electron probe microanalyses (EDS, Oxford X-Max) were exploited to estimate the elemental chemical composition of Ta-N- and Ta-Al-N-based coatings, while an FE-SEM (Sigma Zeiss, Kino, Germany) was used to investigate the surface and cross-sectional morphologies of deposited films. The crystal structure was evaluated by X-ray diffraction (XRD, Philips PW 3710, Amsterdam, The Netherlands) working within Bragg–Brentano geometry and equipped with a Cu-Kα source (40 kV, 30 mA). The collected diffractograms were primarily assessed by means of Match! software v. 3.2.1 (Crystal Impact, Bonn, Germany), to identify the crystalline phases on the basis of the position of detected peaks. Then, profile fits were elaborated by means of Maud software v. 2.79 (L. Lutterotti, University of Trento, Trento, Italy) [34,35], by applying the Rietveld method to the peaks’ broadening and positions [36].

Magnetron-sputtered thin films are usually characterized by residual stress, which must be estimated. Internal stresses within a film can be extrinsic, when induced by phenomena occurring after the deposition process (i.e., due to the film/substrate thermal expansion coefficient mismatch), or intrinsic, when produced during film formation (i.e., due to the grains’ nucleation and growth modes) [37]. A compressive stress state is typical of nitride films deposited via magnetron sputtering techniques, since argon and nitrogen atoms and ions reflected from the target and the sputtered atoms possess high momentum, thus inducing a “peening effect” [38]. When internal stresses are present within the coating, the silicon substrate/coating system undergoes bending deformation in order to reach a mechanical equilibrium. Therefore, from the curvature of the elastically deformed plate-like sample (where substrate thickness is constant and small in comparison to the lateral dimensions) the average film stress, σf, can be estimated in accordance with the Stoney formula [39,40]. This curvature method, together with the Stoney equation, is widely used for the measurement of the residual stresses in coatings deposited by sputtering technologies [41]. The convex or concave curvature of the thin substrates depends on the nature of the coating’s residual stress. Convex curvature is due to compressive stress, with which a negative sign is conventionally associated. Conversely, concave curvature is due to tensile stress, to which a positive sign is attributed.

As already mentioned, in this work, (100) silicon wafers with a thickness ds = 600 μm were used and a biaxial elastic modulus equal to 180 GPa [42] was considered. The curvature analysis was performed by means of stylus profilometry (Dektak XT, Bruker, Billerica, MA, USA).

Hardness (H) and elastic modulus (E) were evaluated via instrumented nanoindentation (NanoTest, Micro Materials, Wrexham, UK), equipped with a sharp Berkovich diamond probe (elastic modulus E = 1140 GPa and Poisson ratio ν = 0.07), calibrated using fused silica and tungsten standards. Tests were conducted regarding depth control, with a maximum penetration depth of 150 nm, establishing that the ratio between the penetration depth (d) and the coating thickness (h) was d/h < 0.1, to minimize the influence of the underlying silicon substrate on the measurements [43]. Statistical analysis was carried out on 25 indentations for each sample, distributed into 5 × 5 grids. The resultant load-displacement curves were elaborated using the Oliver and Pharr method, to estimate the hardness and the elastic modulus of the analyzed surfaces [44,45]. Then, tribological performance indexes, i.e., H/E and H^3^/E^2^, were calculated. The elastic strain to failure, H/E, describes the resistance of the material to elastic deformation, and its importance in wear control has been discussed by Leyland and Matthews [46] and by Lackner et al. [47]. The H/E index can be useful in evaluating the wear properties and tribological performance of coatings [48,49]: a higher H/E value refers to reduced contact pressure, since the applied load is distributed over a larger area and, therefore, a good improvement in wear resistance can be obtained [50]. Recently, it has been progressively accepted that even the elasticity and toughness of the coating are decisive factors in determining its wear resistance, especially in terms of abrasion, impact, and erosive mechanisms, and hardness is no longer the sole and primary requirement [46]. Instead, the H^3^/E^2^ ratio represents the resistance to plastic deformation, and it is related to other near-surface properties, for example, the contact yield pressure and the fracture toughness, since the mechanical behavior of a hard film is strongly affected by the combination of its hardness (H) and elastic modulus (E) [51].

The film-to-substrate adhesion of the Ta-N and Ta-Al-N coatings was investigated with scratch tests, using a UMT-2 tribotester (Bruker), equipped with a standard Rockwell C diamond tip, and the critical loads for failure were then appraised. Test parameters were chosen from the EN 1071-3 standard for ceramic materials in the progressive loading scratch test (PLST) mode [52]. In a scratch test, typical consecutive coating failure events occur at increasing load values, known as critical loads (Lc). It is common to characterize the onset of cracking by the critical normal load Lc1, while the onset of coating spallation is defined as the critical normal load Lc3. The critical loads were assessed by comparing the collected coefficient of friction (CoF) and acoustic emission (AE) signals with SEM images of the produced scratch scars.

Tribological tests were performed using the UMT-2 tribotester, set for pure sliding contact and in dry conditions. The ball-on-flat mode was selected in the sliding tests, in order to achieve high Hertzian contact pressure on coupled surfaces by means of a non-conformal contact type. For wear tests, 5-mm diameter Al_2_O_3_ balls were used as counterparts, supplied by RGPBALLS S.r.l. (Cinisello Balsamo, Italy), with a certified hardness of 1500 HV and produced according to the ASTM F 2094 standard. In each tribological experiment, sliding in a linear reciprocating motion mode was performed, with a path length of 3 mm and a sliding speed of 10 mm/s. The normal load was kept constant in order to obtain 1.3 GPa of initial Hertzian contact pressure [53]. Electron and optical microscopy were used to analyze the topography of the worn surfaces. For each sample, the wear track extent and depth were measured by means of stylus profilometry. The volume of material removed during wear tests was estimated according to ASTM G133-02 [54], wherein the volumetric wear loss could be calculated from the geometrical features of the produced wear tracks. Generally, the wear of ductile materials is dominated by a plasticity mechanism, while in brittle materials, like ceramics, damage occurs through brittle fracture and edge spalling. Both cases can be described using Archard’s law [55], which correlates the worn volume with the hardness of the sliding surfaces, the applied load, and the overall sliding distance. Archard’s model is described by Equation (1) and is normally exploited for evaluating sliding wear damage:

(1) $$K=\frac{\Delta V\cdot H}{F_{N}\cdot L}$$

where *K* is the dimensionless generalized wear coefficient, Δ*V* is the worn volume (m^3^), *L* is the sliding distance (m), *F_N_* is the normal contact force (N), and *H* is the hardness (Pa) of the surface material that is worn away. It is a property of the coupled materials system, which depends on the surface morphological characteristics, sliding conditions, and environment. Sometimes, a more convenient and physically direct quantity with respect to *K* is the “specific wear rate”, k, which is normally given with specific dimensions (10^−6^ mm^3^/Nm) and represents the worn volume divided by the mechanical energy input into the contact. In this research work, k was estimated for all deposited coatings.

Raman spectra were acquired using an InVia µRaman instrument (Renishaw, Wharton Underage, UK) exciting at 488 nm (30 mW nominal power) and using a 20× objective. Baseline subtraction was performed with WiRE software v.4.4 (Renishaw, Wharton Underage, UK), whereas Matlab v. R2020b (MathWorks, Portola Valley, CA, US) was used for µRaman maps reconstruction. Spectra are stacked for clarity in the figures. Acquisitions outside the scratch were used to account for the unperturbed Ta-N and Ta-Al-N film, whereas the spectra identified as worn were recorded over the scratch. More than 50 spectra that were recorded at different positions inside the wear scars and were then averaged.

## 3. Results and Discussion

As reported in Table 1, two series of samples were obtained by changing the substrate bias from −50 V to −100 V and using a gas flow ratio of N_2_:Ar = 1:1. 

**Figure 2:**
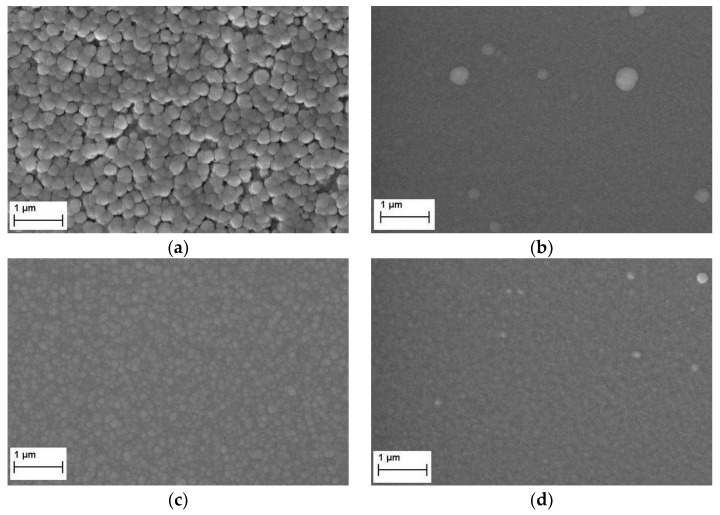

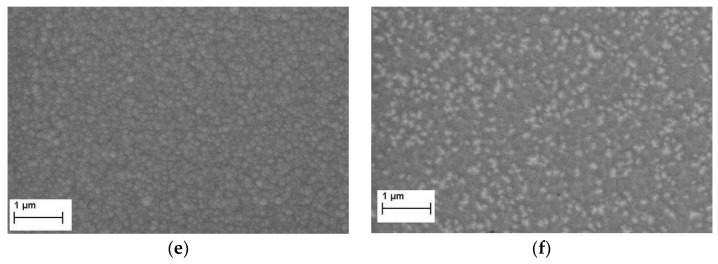
SEM images of coatings deposited with different bias values: (**a**) TaN1 −50 V, (**b**) TaAlN1 −50 V, (**c**) TaN2 −75 V, (**d**) TaAlN2 −75 V, (**e**) TaN3 −100 V, (**f**) TaAlN3 −100 V.

In SEM investigations, all tantalum nitride deposited coatings (except for sample TaN1) appear dense and compact, with a granular surface topography (see Figure 2). The Ta-N coating deposited at −50 V, visible in Figure 2a, shows several crevices between the granular structures. Comparing the SEM top views in Figure 2a,c,e, dome-shaped features are clearly visible, but their sizes decrease as a function of the applied bias voltage. Indeed, the spherical-topped particle average diameter changes from (187 ± 14) nm for TaN1 to (132 ± 10) nm for TaN2, to (113 ± 7) nm for TaN3. As reported in the literature, a bias voltage rise improves the adatoms’ mobility during the film growth due to the energetic particle bombardment process, which results in the removal of asperities, voids, and defects in the material, leading to smoother surfaces [29].

In the case of the Ta-Al-N samples (Figure 2b,d,f), the surfaces appear more uniform and the granular appearance that is typical of Ta-N films is much less obvious. Looking at the TiAlN1 sample (Figure 2b), the presence of some macroparticles can be observed; in the case of TaAlN3 (Figure 2f), deposited at −100 V bias, the surface shows some facetted crystallites [56].

**Figure 3:**
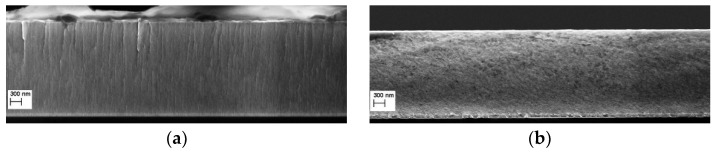
Fracture cross-sectional micrographs of (**a**) TaN2 and (**b**) TaAlN2 films, deposited using a bias voltage equal to −75 V.

Figure 3 shows an example comparison of the fracture cross-sectional micrographs of the TaN2 and TaAlN2 films, deposited using a bias voltage equal to −75 V. Keeping the deposition conditions unchanged, in the case of the tantalum nitride sample, a columnar growth is quite evident, which instead disappears with the addition of aluminum. This leads us to assume a granular growth that is likely due to the incorporation of Al atoms into the Ta-N phase. The growth rate of deposited Ta-N films is about 7 nm/min, while it grows up to 17 nm/min for Ta-Al-N coatings; therefore, the deposition durations were adjusted to gain overall coating thicknesses of around 2.5 μm.

From the X-EDS analyses, the N/Ta and N/(Ta + Al) atomic ratio in various coatings grown at different bias values resulted in over-stoichiometry for all samples, as reported in Table 1. The deviation from the nominal cathode composition and the incorporation of excess N within the deposited films may be related to the formation of N-rich phases and have been already observed [56,57]. For the Ta-Al-N sample series, the N content was found to slightly decrease as the bias voltage increased up to −100 V.

In order to investigate the nitride phases in the deposited materials, XRD diffractograms were evaluated.

**Figure 4:**
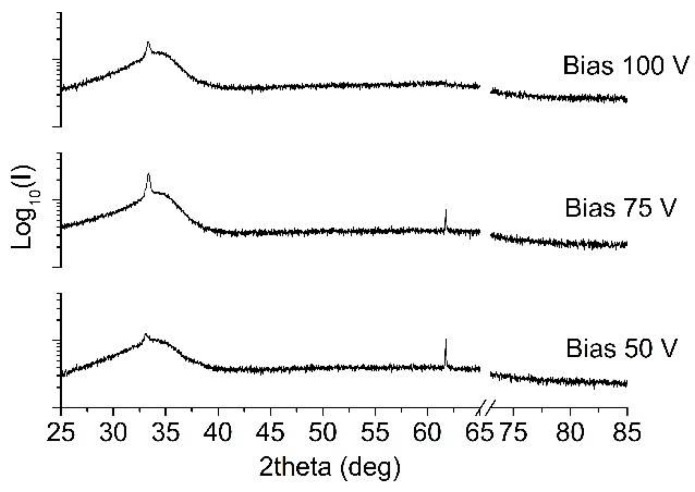
XRD patterns of TaN films deposited at different bias voltages.

The X-ray diffraction spectra of Ta-N-based coatings were not identifiable with a particular crystal structure (Figure 4). Only a single broad peak without any other signal could be detected, spectra collected in grazing incidence mode also revealed the presence of the same very broad peak, centered at around 35°, having a width of over 7°. This could be assigned to the (111) plane of cubic *fcc* B1 δ-TaN phase (ICDD 49-1283), as already identified by Elangovan et al. [58] and Tsukimoto et al. [59]. The broad feature in the collected patterns and the absence of other diffraction peaks suggest that the coatings were either partially amorphous or highly disordered, with a low degree of crystallinity and fine grain size. The detected predominant cubic phase within the sputtered Ta-N films is in agreement with previous results reported by Lee et al. [57] and Stavrev et al. [60] for high N_2_/(Ar + N_2_) flow ratios during sputtering. It has been observed that in over-stoichiometric nitride coatings, excess nitrogen atoms can be located randomly in tetrahedral sites, thus resulting in a certain amorphization of the coating structure [61]. No significant differences could be noticed among the various coatings deposited at increasing bias values.

Despite the observed over-stoichiometry, the detected broad peak is not compatible, neither with hexagonal Ta_5_N_6_ nor with monoclinic Ta_3_N_5_, which are recognized to be the N-richest Ta-N phases and that have been detected for [N] ≥ 35% [62]. In Figure 4, the breakage in the *x*-axis allows the suppression of the strong signal coming from the underlying Si substrate, thus clarifying the readability of the spectra.

**Figure 5:**
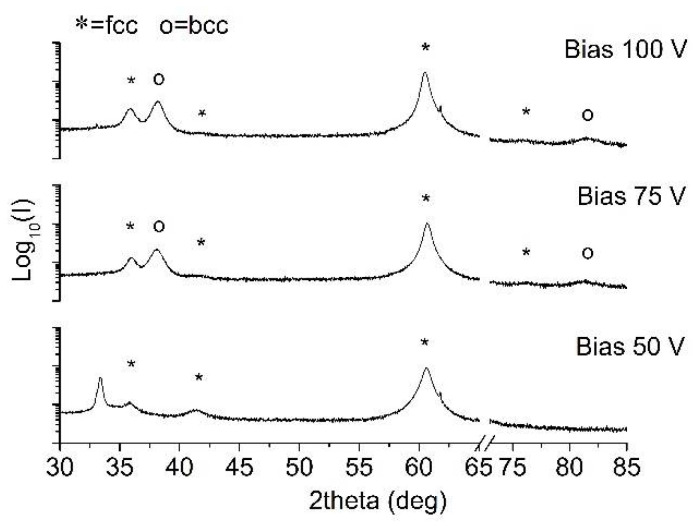
XRD patterns of TaAlN films deposited at different bias voltages.

In Figure 5, regarding XRD diffractograms of Ta-Al-N-based coatings, the main reflections of a single phase that is compatible with a B1 cubic solid solution, having a lattice parameter of a = 4.339 Å [58], are reported. All the deposited coatings appeared to consist predominantly of a supersaturated *fcc* δ-TaN-type Ta_1−x_Al_x_N solid solution [63,64,65]. No distinct AlN peaks were visible, implying that the coatings predominantly consist of a Ta-Al-N solid solution. The collected spectra show diffraction peaks at 2θ angles of 35.5°, 41.3°, and 60.1°, which can be identified as the (111), (200), (220) peaks, respectively, of the *fcc* lattice. According to the Ta–N phase diagram, δ-TaN is a metastable phase at room temperature, but it often appears during sputtering deposition [24]. Small additions of Al into Ta-N have been proven to stabilize the δ-TaN phase [6,64]. For the growth of transition metal nitrides by reactive sputter deposition, the interaction and competition among different interconnected surface reactions and diffusional processes govern film growth kinetics, surface morphology, film microstructure, and texture. In this case, texture development is competitive with both (111) and (220) grains nucleating at first. In line with the bulk phase, selected as a reference, the strongest diffraction line should be (111), since it represents the plane with lower surface energy, thus, being favored. However, the relative intensities of the detected peaks changed significantly with respect to the line, leading us to infer the presence of a different texturing effect, as already observed by Koller et al. [25], due to the greater availability of energy provided by the HiPIMS deposition process. In fact, the HiPIMS process promotes the adatoms’ mobility and diffusional processes, which are sufficient to develop a different crystal orientation at the expense of that of (111) [9]. The texture coefficients, *T*, reported in Table 2 were determined from the Bragg–Brentano (θ–2θ) scans and can be defined using Equation (2):

(2) $$T\left(hkl\right)=\frac{I\left(hkl\right)}{I\left(111\right)+I\left(200\right)+I\left(220\right)}$$

where *I*(*hkl*) represents the measured peak intensities for the (111), (200), and (220) reflections. It can be observed in Table 2 that all the produced films exhibit a predominant (220) orientation, since the T_(220)_ value is significantly higher than that of the reference phase, starting from the sample produced with the lowest bias, and tends to increase progressively, even if slightly, revealing that the identified texture is typical of the process conditions. Correspondingly, the value of T_(200)_ remains close to zero and faintly decreases. The free energy, resulting from the competition between the strain energy on different lattice planes and the surface energy, determines the preferred orientation of the deposited films. Therefore, this is related to surface energy, adatom mobility, and ion bombardment, which are strongly enhanced in the HiPIMS deposition process compared to conventional magnetron sputtering technologies [66].

The position of detected peaks appeared to have shifted because of crystalline lattice deformations, which are generally associated with two phenomena occurring in sputtered coatings: deformation by solid solution, due to the incorporation of atoms within the crystal lattice, and deformation by residual stresses, which is typical of the deposition process [67]. In the collected spectra, the shift to higher diffraction angles can be attributed to a decreasing lattice parameter, similar to that also observed by Schalk et al. [63], since the smaller Al is a substitute for Ta in the *fcc* Ta-N lattice, as reported in Table 2.

The peak broadening visible in Figure 5 confirmed the grain refinement induced by the deposition methodology. From the reported data, it can be seen that a new reflection appeared at a bias higher than 50 V, located at 2θ = 38.4°. This could be attributed to a single-phase body-centered cubic Ta structure with the space group Im-3m [68], containing a small amount of dissolved nitrogen in the interstitial voids of the metal structure [69], since the main peak (110) of the bulk Ta should be located at 2θ = 38.3°. A second signal in the same structure can be clearly seen at 2θ = 81.9°, which refers to reflection (220), thus suggesting that the applied bias promotes the formation of two distinct phases.

The bias voltage during the coating deposition process is an influential parameter since it affects the energy of the impinging ions. With increasing bias, enhanced adatom mobility is induced, caused by the improved energy of the ion bombardment and it realistically affects the final structure of the growing material, including the mean grain size, which appeared larger at higher applied bias.

**Figure 6:**
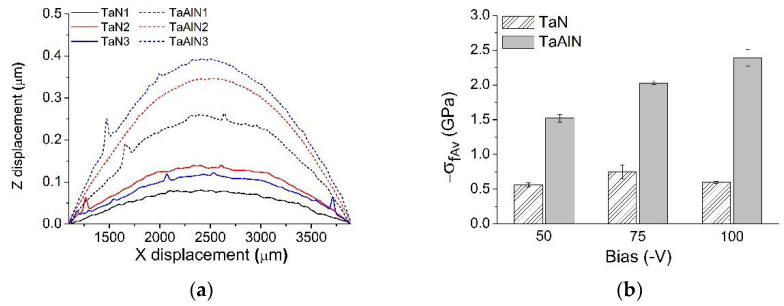
Residual stresses of the TaN and TaAlN coatings, measured by (**a**) the curvature method and (**b**) the Stoney equation.

For the deposited Ta-N and Ta-Al-N films, it can be clearly observed in Figure 6a that they all exhibit a convex curvature associated with compressive stresses, which appeared more pronounced for those samples containing Al.

Figure 6b shows residual stress values estimated with the Stoney equation. Ta-Al-N coatings show a monotonically increasing residual stress with increasing bias. Conversely, Ta-N coatings do not show a precise trend, and their relatively low residual stress values remain well below −1.0 GPa. Therefore, it appears that the addition of Al induced the development of higher residual stress, probably arising from the lattice distortion due to Ta substitution with the smaller Al species. In the plane of the film, however, the film is not free to expand; thus, macroscopic compressive stresses arise.

The monotonous increase in stress, observed for Ta-Al-N coatings as the bias grows, comprises both the contributions of extrinsic and intrinsic stresses. Thermal stress, *σ_th_*, can be calculated using Equation (3) [38]:

(3) $$\sigma_{th}=\frac{\left(\alpha_{s}-\alpha_{f}\right)}{1-\nu_{f}}E_{f}\Delta T$$

where *α_s_* and *α_f_* are the coefficients of thermal expansion of the substrate and the film, respectively; *E_f_* and *υ_f_* are the elastic modulus and Poisson’s ratio of the coating; Δ*T* is the temperature variation during the deposition process. As reported in the literature [70], it is possible to assume that when changing the bias voltage from −50 V to −100 V, the average temperature increases (thanks to the higher heat flux), and the coating elastic modulus improves (see the following). Therefore, the product *E_f_*Δ*T* (i.e., the thermal stress) rises as a consequence. Concerning intrinsic stresses, the substrate bias voltage variation intensifies the so-called ‘‘peening effect’’, affecting the adatoms’ mobility and cluster formation, thus enhancing the nucleation rate and compressive stress.

Concerning the Ta-N coatings, as mentioned above, it is not possible to observe a similar stress value linear dependence on the applied bias voltage, perhaps due to their partially amorphous character.

**Figure 7:**
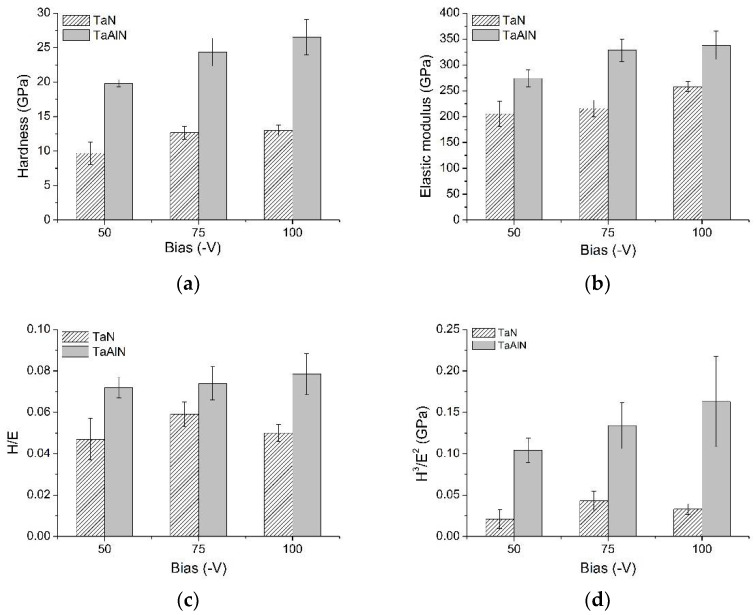
Mechanical properties, as estimated from nanoindentation measurements: (**a**) hardness, (**b**) elastic modulus, (**c**) elastic strain to failure H/E, and (**d**) resistance to plastic deformation H^3^/E^2^.

Generally, high compressive residual stress accounts for high hardness [71], as can be seen in Figure 7, where surface mechanical properties by nanoindentation are reported.

At the same deposition conditions, the introduction of Al implies an evident increment of the mechanical properties. The hardness almost doubles, changing from a minimum value of ~9.7 GPa for TaN1 (bias voltage = −50 V) to a maximum value of ~26.5 GPa for TaAlN3 (bias voltage = −100 V), while the elastic modulus of Al-containing films is also improved, being within the range of 275–340 GPa.

The reasons for this enhancement of mechanical features are several. On the one hand, Al atoms partially replace the Ta ones, forming a substitutional solid solution, then both a refinement of Ta-N crystal structure and a solid-solution strengthening effect [72] occur, leading the films to develop better mechanical properties.

On the other hand, the hardness increase can also be attributed to both the residual stress and the higher density of Ta-Al-N products, which is detectable by the improvement of the elastic modulus in these films. Moreover, Ta-N coatings, presenting only the (111) peak of the corresponding *fcc* microstructure and a partially amorphous structure, show half the hardness of Ta-Al-N films, with a (220) texturing feature. According to Tan [9] and Shin [73], the (111) reflection is related to an under-dense layer with voids within its microstructure. Therefore, the incorporation of a third element into tantalum nitride coatings, forming a ternary nitride, results in coatings exhibiting enhanced mechanical properties in comparison with binary nitride coatings.

For all films, while increasing the applied bias voltage, it is possible to observe a corresponding slight improvement in both hardness and elastic modulus that are generally correlated, as shown in Figure 7a,b. The enhanced density with the applied bias that has already been observed in morphological characterization could inhibit the mobility of the defects, and a stronger mechanical behavior might be expected.

In Figure 7c,d, H/E and H^3^/E^2^ are also presented. From a purely mechanical point of view, the increased H/E ratio of the Ta-Al-N coatings with respect to Ta-N should indicate the good ability of Al-containing films to withstand mechanical weakening in tribological contact service, since the contact remains longer within the elastic field. At the same time, the high H^3^/E^2^ ratio suggests a possible improved ability of the material coating to bear impact energy from deformation to fracture [26].

**Figure 8:**
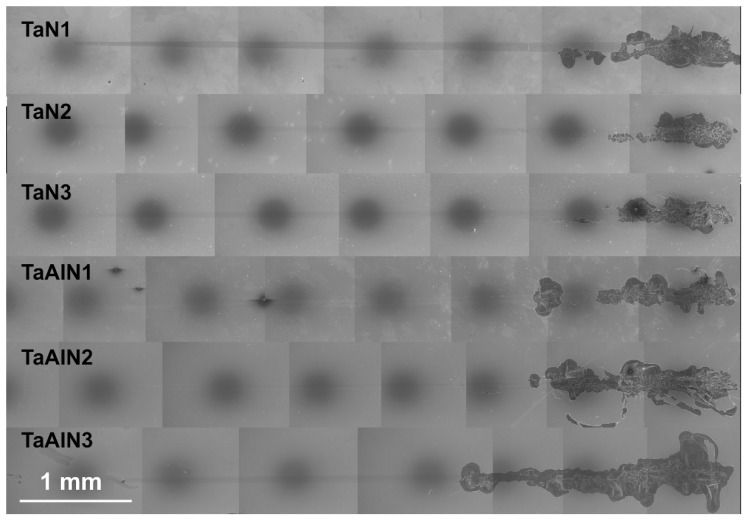
Scratch scars of TaN and TaAlN coatings.

Scratch tests were performed to investigate the film/substrate adhesion and interface strength. The films’ failures are reported in Figure 8.

The applied normal load was increased up to 60 N, according to the EN 1071-3 standard, in order to reach the Lc3 critical load, representing the complete delamination of the coatings.

Although the two coating types exhibit different mechanical properties, almost all coatings start to be damaged at applied vertical loads of between 33 and 37 N (see the Lc1 values in Table 3). Moreover, from an observation of the scratched surfaces, it appears that the subsidence of the substrate occurs together with the coating failure. More precisely, the progressive breakdown of the interface and the subsequent film delamination are due to the breakdown of the underlying silicon. The scratch scars were observed by electron microscopy, and the collected pictures highlight that Ta-N samples exhibit higher scratch resistance than those of Ta-Al-N, for which complete delamination occurs at lower applied loads, as is visible in Figure 8. Among the Ta-N films, the worst behavior is observed in the TaN1 sample, due to its coarser microstructure, higher roughness, and minor cohesion of the grains forming the film.

The different behaviors between the two coating types could be ascribed to the higher residual stress level in the Al-containing films and to the better coupling of the elastic modulus between the coatings and silicon substrate in the case of the Ta-N films. Among the Ta-N films, the poorest behavior is exhibited by the TaN1 sample, probably because of its coarser microstructure, higher roughness, and minor cohesion of the grains forming the film.

In all samples, the adhesion is strong: up to Lc1 values, the traces are all smooth and there are no signs of damage. The films remain intact and seem to accommodate the deformations imposed by the passage of the tip. The fracture modes are similar for all specimens, which fail by traction: on all scratched surfaces, forward-chevron cracks can be observed at the borders of the scratch track. Then, mixed forward-chevron tensile-type and Hertzian cracks within the track appear, with local interfacial spallation at the borders. Even when fractured, the coatings remain adhered to the substrate and, at higher loads when substrate failure occurs, gross interfacial shell-shaped spallation appears along the outer edges of the tracks.

When failure occurred, the coatings underwent chipping failure, and the breakage mode appeared brittle since delamination with substrate exposure and without plastic deformation could be observed. The critical loads of Lc1, estimated from image analyses and tribological data elaborations, are reported in Table 3, corresponding to crack initiation, as estimated by the AE onset and the corresponding sudden change in CoF. Conversely, it was not possible to estimate the Lc3 values since they were not associated with film/substrate adhesion but rather with the failure of the silicon substrate.

The obtained results also demonstrate the strong role played by compressive residual stresses in establishing susceptibility to catastrophic film fracture.

Wear tests were conducted in the reciprocating linear motion mode on each sample surface. Despite the fact that the mechanical properties were clearly inferior, Al-free coatings exhibited higher wear resistance, characterized by low material removal rates. Therefore, in the case of the Ta-Al-N coatings, after 1100 cycles, wear grooves of over one micron deep were measured, while for the better-performing Ta-N films, it was necessary to increase the number of cycles up to 5000 to obtain geometrically evaluable wear scars. The tribological data are summarized in Table 3. For the TaN1 sample, the less dense and compact structure proved to be unsuitable to withstand the stresses imposed during the wear test: the coating prematurely underwent catastrophic breakage, preventing a plausible estimate of the wear rates themselves; thus, it has not been considered in this context.

The variations of CoF with sliding cycles for different coatings have a similar general trend and remain at a relatively stable level after a short run-in time. CoF stabilizes at the same value within the Ta-Al-N samples, with a slight increase due to the applied bias voltage, a sign that for this material, the friction phenomena are little affected by this parameter. Instead, there is a difference between TaN2 and TaN3 samples, since the TaN2 coating exhibits a slightly lower friction coefficient.

Even the mechanism and dynamics of wear are significantly different between the two types of coating. As mentioned above, Ta-Al-N coatings appear more prone to wear damage, while Ta-N films show better wear resistance. Within each group, no great differences in wear behavior could be observed, due to the varying applied bias voltage.

Since no wear-trace depth exceeded the minimum thickness of the produced films, i.e., 2.5 µm, it can be established that no film breakage occurred during the tribological experiments, nor was the substrate ever exposed. Thus, the mean wear rate of the Ta-N and Ta-Al-N coatings could be estimated from the geometrical features of the wear scars.

**Figure 9:**
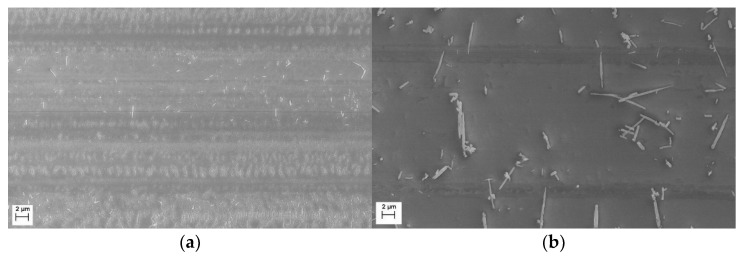
Morphology of the bottom of the wear track on samples TaN3 (**a**) and TaAlN3 (**b**).

In Figure 9, the surface morphologies within wear scars on samples TaN3 and TaAlN3 are reported. It is clear that the Ta-N coating underwent plastic deformation at the wear surface, with slightly adhesive wear similar to what was observed by Tan et al. [9], and delamination by fatigue is the dominant wear mechanism. Otherwise, the wear mechanism on the Ta-Al-N material looks completely different since no abrasion or delamination phenomena can be observed. The formation of wear debris takes place, wrapping around itself as if to form small rolls, and the wear process generally results in the smoothing of the worn surface [74]. Rolls tend to be aligned perpendicularly to the sliding direction. Similar rolls can also be detected on the Ta-N worn surfaces, but they are very limited in number and size.

The variances in morphology may be due to the different resistance to plastic deformation, i.e., H^3^/E^2^, within the two types of films. The Ta-N material is probably more likely to deform plastically under the action of the applied load, with peeling-off taking place within the wear trace, visible as small cracks/fine lines that are orthogonal to the sliding direction, as shown in Figure 9a.

The calculated specific wear rate is reported in Table 3: a meaningful difference can be observed between the two coating types since the k values differ by one order of magnitude, with the Ta-N samples exhibiting the highest wear resistance. Despite their superior mechanical properties, Al-containing films are more sensitive to wear phenomena and wear out rapidly: this suggests that the reason for this can be attributed to the different wear mechanisms that are activated on the different types of films. In fact, within the same wear mechanism, materials with better mechanical properties and, in particular, with a higher H/E index, should perform better. However, this is not the case.

The estimated wear rates of the Ta-N samples can be considered within the mild wear regime. Furthermore, the observation of surface smoothing by the wear process is a further feature of mild wear, wherein the asperities on the as-deposited coating surface (Figure 2c) are removed in a uniform and stable way, leaving a flat surface afterward, in comparison to that observed in SEM images, wherein dome-shaped features are clearly visible.

This regime is generally accompanied by the development of a tribofilm, consisting of constituents from both sliding surfaces, i.e., the sample and the counter-body. Then, material transfer occurs, resulting in an oxidized tribofilm that is usually made of elements from both surfaces [75]. Among the different phases that can form, there are Magnéli phases containing crystallographic shear planes, which are known to endorse low friction [76].

In fact, the X-EDS analyses from the abrasion region of TaAlN3 film indicate that the surface material was oxidized since a large amount of oxygen could be detected. Although the X-EDS results indicate that O is present in the wear debris, in addition to the constituents of the coating, no friction reduction could be observed for Ta-Al-N coatings; therefore, it can be assumed that no Magnéli-type phases are formed in this instance but, rather, amorphous ones.

**Figure 10:**
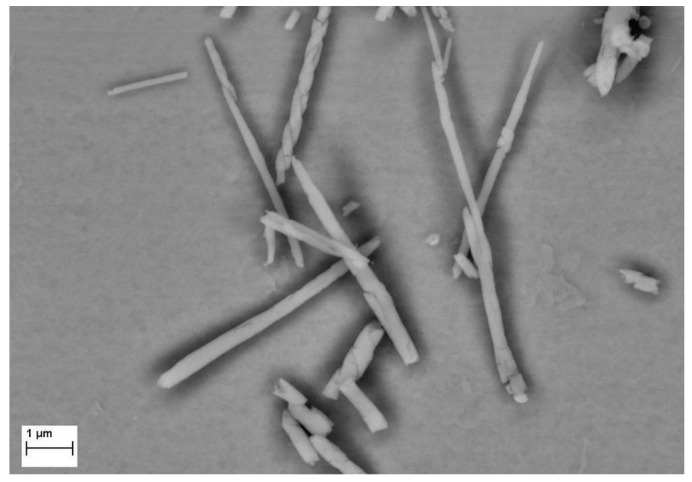
Detail of wear debris on the worn surface of sample TaAlN3.

A large number of small, rolled sheets can be detected on the worn surface of the Ta-Al-N sample, as shown in Figure 10.

The oxidized material forming the wear debris comes from the worn coating since no oxygen could be detected within the wear scar apart from the debris itself. The debris appears as small sheets rolled up around themselves to form micrometric rolls, revealing a certain ductility. Similar roll-like debris has already been observed by other authors on hard ceramic coatings [66,74,77] and has been associated with severe friction. Rainforth [77] used TEM to demonstrate that the rolls are amorphous and come from the mild wear of tetragonal zirconia worn against alumina, while Yang et al. [78] observed roll-like wear debris on a Ti-Mo-N coating worn against tungsten carbide (WC. These rolls could only be observed during the sliding of hard materials in the mild wear regime and are due to the formation of a tribofilm. Hence, they are expected to contain elements from the surrounding environment.

The roll formation is probably associated with the plastic deformation of the tribofilm at the interface between the film and the counter-body during the wear processes. Their greater inclination toward surface oxidation makes the Ta-Al-N coatings more prone to the formation of such debris and, consequently, less wear-resistant than Ta-N ones. Thus, Ta-Al-N films are more susceptible to wear phenomena because of the continuous removal and reformation of the oxidized tribofilm.

**Figure 11:**
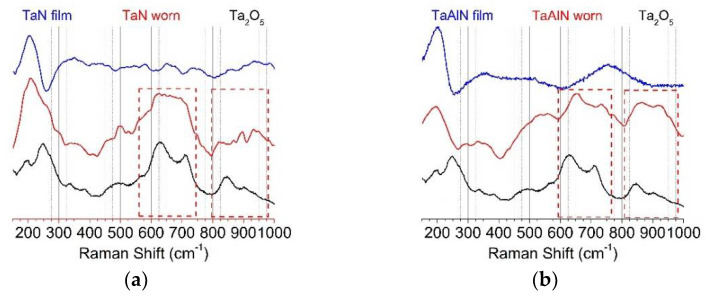

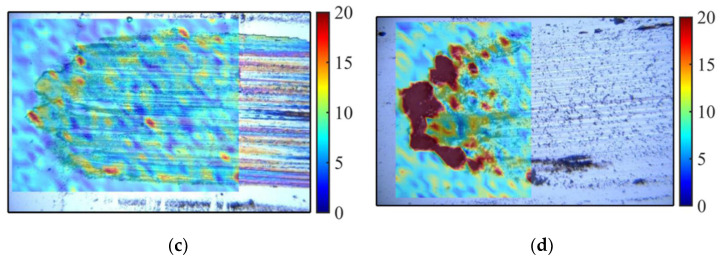
Raman spectra of (**a**) TaN3 and (**b**) TaAlN3 coatings for the as-deposited and worn films compared to the spectrum of Ta_2_O_5_. The most characteristic bands of Ta oxides are highlighted in the red boxes. (**c**) Ta-N and (**d**) Ta-Al-N film µRaman maps on the scratched area, reporting, in false colors, the Raman intensity of the band of Ta oxide at 650 cm^−1^.

In order to better understand the nature of the tribofilm, micro-Raman measurements were carried out on the worn surfaces. A comparison between the as-deposited and worn coatings is shown in Figure 11.

Spectra of the as-deposited films (blue lines) reveal the presence of the characteristic longitudinal acoustic (LA) mode of Ta-N_x_ at about 200 cm^−1^ [79,80].

A Raman spectrum of Ta_2_O_5_, used as a reference in Figure 11a,b (black line), shows the Raman bands associated with the oxide at about 650 and 900 cm^−1^, which can be assigned to the Ta-O stretching and bending modes. These bands can be clearly seen in the spectra collected inside the worn areas (red lines) of both the Ta-N (Figure 11a) and Ta-Al-N films (Figure 11b). Micro-Raman maps of the wear tracks are reported in Figure 11c,d using the intensity of the Raman signal of the Ta oxide at 650 cm^−1^. The maps clearly show that the worn structures observed in the SEM images with an increased content of oxygen are due to Ta oxides. The maps also show that the Ta-N_x_ film has a superior adhesion strength and minimal inclination to plastic deformation since, in the worn area of this film, there are only small regions that show the presence of Ta oxides, whereas large structures are observed in the maps of the Ta-Al-N film.

## 4. Conclusions

In this work, Ta-N and Ta-Al-N coatings were deposited using HiPIMS technology for protective purposes. The effects of both introducing Al in order to form a ternary nitride and also changing the applied bias voltage have been investigated in terms of microstructure and mechanical and tribological behavior. The comparison between the two coating types revealed that aluminum effectively changed the microstructural features of films, which exhibited a changed preferred orientation, higher crystallinity, and increased residual compressive stress. In addition, the mechanical properties were strongly affected: (i) the introduction of aluminum within the Ta-N reticule conferred significantly higher mechanical characteristics; (ii) the measured H and E underwent an improvement, with increasing bias voltage in both the Ta-N and Ta-Al-N coating types. In fact, the maximum hardness value of 26.5 GPa was obtained for the Ta-Al-N film, produced with the highest applied bias voltage of −100 V, while for the corresponding Ta-N film, the recorded hardness was 13 GPa. Both the residual stress state and the crystalline microstructure, as well as the solid-solution hardening phenomenon due to the incorporation of Al atoms within the Ta-N reticule, contribute to the increase in the mechanical properties of the Ta-Al--N films. In addition, all the deposited coatings demonstrated excellent adhesion to the Si substrate. For all of the samples, a cohesive through-thickness cracking failure due to tensile stress was observed only at a high normal load (Lc1 > 30 N).

Despite their superior mechanical properties, the Ta-Al-N coatings revealed high wear coefficients with respect to Ta-N films, ascribable to a different wear mechanism. Both the increased residual stress level and the variable wear mode contributed to worsening the wear resistance of Ta-Al-N films (dry conditions, room temperature, and an Al_2_O_3_ counter-body). Under the test settings considered in this work, the Ta-N coating underwent plastic deformation at the wear surface, with slightly adhesive wear. However, no abrasion or delamination phenomena could be observed on the Ta-Al-N film with the formation of roll-like wear debris, which was probably associated with the plastic deformation of an oxidized surface tribofilm during the wear process. The greater inclination to surface oxidation makes the Ta-Al-N coatings more prone to the formation of such debris; consequently, they are more susceptible to wear phenomena because of the continuous removal and reformation of the oxidized tribofilm. Micro-Raman tests confirmed that in the worn areas of the Ta-N film, only small oxidized regions were revealed, whereas wider areas could be observed in the maps of the Ta-Al-N films.

## Figures and Tables

**Table 1:** Deposited samples studied in this work.

Sample	Target	Bias (V)	Deposition Rate (nm/min)	N% at.	Al% at.	Ta% at.
TaN1	Ta	−50	7.1	61.7 ± 0.8	/	38.3 ± 0.8
TaN2		−75	6.6	62.6 ± 0.5	/	37.4 ± 0.5
TaN3		−100	7.4	62.5 ± 0.9	/	37.5 ± 0.9
TaAlN1	TaAl	−50	16.7	61.0 ± 0.9	16.5 ± 0.4	22.5 ± 0.5
TaAlN2		−75	16.0	59.7 ± 0.2	17.1 ± 0.1	23.1 ± 0.2
TaAlN3		−100	17.3	58.9 ± 0.7	17.4 ± 0.3	23.6 ± 0.4

**Table 2:** Microstructure parameters estimated from XRD analyses.

Sample	Bias (V)	*a* (Å)	Av. Cryst. Size (nm)	T_(111)_	T_(200)_	T_(220)_
TaAlN1	−50	4.327	13 ± 1	0.11	0.06	0.83
TaAlN2	−75	4.320	15 ± 1	0.11	0.04	0.85
TaAlN3	−100	4.326	22 ± 2	0.10	0.02	0.88
Ref. (ICDD 49–1283)	-	4.339	-	0.49	0.32	0.19

**Table 3:** Critical loads in scratch tests and tribological data after wear tests of Ta-N and Ta-Al-N coatings.

Sample	Bias	Scratch	Wear	
		Lc1	CoF	Specific Wear Rate k
	(V)	(N)		10^−6^ mm^3^/Nm
TaN1	−50	34 ± 5	Not Evaluable	Not Evaluable
TaN2	−75	34 ± 2	0.61 ± 0.11	2.45 ± 0.07
TaN3	−100	36.8 ± 0.7	0.70 ± 0.12	2.28 ± 0.04
TaAlN1	−50	34.3 ± 0.4	0.69 ± 0.12	54.0 ± 0.3
TaAlN2	−75	33 ± 1	0.71 ± 0.11	46.3 ± 0.1
TaAlN3	−100	33.1 ± 0.8	0.73 ± 0.13	44.0 ± 0.4

## Data Availability

Data are available on request due to privacy restrictions. The data presented in this study are available on request from the corresponding author.
